# Muscular profiles of adolescent female soccer players: a cross-sectional comparison of hamstring-to-quadriceps and vastus medialis-to-vastus lateralis characteristics

**DOI:** 10.1186/s13018-026-07063-0

**Published:** 2026-06-23

**Authors:** Sonoka Ichikawa, Mikuri Tanaka, Rina Watabe, Ikumi Morikawa, Daigo Matsumoto, Kaede Mada, Ryota Akagi

**Affiliations:** 1 Graduate School of Engineering and Science, Shibaura Institute of Technology, 307 Fukasaku, Minuma-ku, Saitama-shi, 337-8570 Japan; 2 College of Systems Engineering and Science, Shibaura Institute of Technology, 307 Fukasaku, Minuma-ku, Saitama-shi, 337-8570 Japan

**Keywords:** Hamstring-to-quadriceps ratio, Isometric peak torque, Isotonic peak power, Knee extensors, Knee flexors, Muscle stiffness

## Abstract

**Background:**

To characterize multifaceted muscular profiles potentially associated with knee joint sports injuries in adolescent female athletes, we compared hamstring-to-quadriceps (H:Q) ratios derived from isometric torque and isotonic power, as well as the relative muscle quality characteristics of the vastus medialis (VM) and vastus lateralis (VL), between adolescent female soccer players and recreationally active controls.

**Methods:**

In this cross-sectional study, 97 adolescent females (soccer: *n* = 62; control: *n* = 35) were categorized into older (≥ 162 months) and younger (< 162 months) cohorts. Assessments of both legs included isometric peak torque and isotonic peak power of the knee extensors and flexors using a dynamometer, along with ultrasound-derived muscle echo intensity and shear modulus of the VM and VL at rest. The H:Q ratios were calculated from both torque and power, whereas VM:VL ratios were derived from echo intensity and shear modulus to evaluate relative muscle quality characteristics.

**Results:**

The isometric H:Q ratio was significantly higher in the soccer group than in the control group (pooled across age groups and legs; soccer: 55.3% ± 9.8%; control: 51.0% ± 10.4%). However, no significant between-group differences were observed in the isotonic H:Q ratio (soccer: 86.3% ± 21.1%; control: 81.4% ± 27.9%) or in the VM:VL ratios for echo intensity (soccer: 91.0% ± 10.4%; control: 91.8% ± 7.2%) and shear modulus (soccer: 84.0% ± 22.8%; control: 83.9% ± 23.2%). Furthermore, the isometric H:Q ratio showed no significant effects for age or leg dominance, whereas the isotonic H:Q ratio was significantly higher in younger participants and in the non-dominant leg.

**Conclusions:**

These findings suggest that isometric and isotonic H:Q ratios capture distinct aspects of knee muscle function. Additionally, no significant differences were observed between elite soccer players and recreationally active peers in the isotonic H:Q ratio or ultrasound-derived VM:VL ratios, suggesting that highly trained adolescent female soccer players do not necessarily exhibit distinct dynamic power or resting muscle quality. These multifaceted muscular profiles may provide exploratory laboratory-based reference data, although longitudinal studies are needed to determine how these characteristics relate to actual injury incidence.

**Supplementary Information:**

The online version contains supplementary material available at https://doi.org/10.1186/s13018-026-07063-0.

## Background

Anterior cruciate ligament (ACL) injuries represent one of the most significant orthopedic challenges in sports medicine, with female athletes experiencing a two- to eight-fold higher incidence than their male counterparts [1]. Among various sports, female soccer has been identified as a particularly high-risk activity [2], with an especially high prevalence observed among adolescent players [3]. These injuries are often associated with reduced athletic performance and shortened career longevity [4, 5]. Furthermore, because an initial ACL injury significantly increases the risk of subsequent re-injury [6], primary prevention in adolescent female soccer players is essential for long-term athletic success.

The hamstring-to-quadriceps (H:Q) torque ratio is a widely recognized indicator of strength balance between the antagonistic muscles of the knee [7, 8]. Traditionally, this ratio has been used to monitor functional recovery in patients with ACL deficiency [9] or reconstruction [10–12]. However, it may also represent a muscle characteristic potentially associated with ACL injury-related neuromuscular profiles because the ratio reflects the critical relationship between quadriceps-induced anterior tibial shear force and the hamstrings’ ability to counteract this force through posterior tibial shear [13]. Although the H:Q ratio has been proposed as a predictor of ACL injury risk, evidence supporting its predictive value remains inconsistent [7], potentially owing to methodological differences among studies [14]. While the functional H:Q ratio (eccentric hamstring to concentric quadriceps strength) has been suggested as a more representative measure, its assessment is largely limited to constant-velocity isokinetic conditions [15]. In reality, athletic maneuvers involve highly acceleration-dependent, isotonic contractions. Therefore, evaluating conditions that better reflect the physiological power output during athletic maneuvers may provide a more comprehensive understanding of knee muscular profiles. To address this gap, assessment of the H:Q ratio based on isotonic power may offer a novel, hypothesis-driven perspective on muscular characteristics potentially associated with knee joint sports injuries. This approach is consistent with recent calls for injury prevention strategies that better simulate real-world athletic demands.

The relationship between components of the quadriceps femoris, particularly the vastus medialis (VM) and vastus lateralis (VL), is considered another critical neuromuscular feature potentially associated with knee joint loading patterns. Although quadriceps force generally contributes to ACL loading [16, 17], musculoskeletal modeling has demonstrated that the VL exerts the greatest influence on ACL loading, producing approximately five times greater ACL load than other individual quadriceps muscles during stop-jump tasks [18]. While the VL is a primary driver of ACL tension, the VM plays an important stabilizing role. VM activation is particularly prominent during weight-bearing tasks that challenge knee stability, highlighting its role in maintaining proper joint alignment [19]. Supporting this, a previous study reported that a greater VM to VL muscle thickness ratio is associated with lower knee valgus and transverse plane knee moments during single-leg landing tasks, both of which are biomechanical parameters linked to ACL injury risk [20]. Collectively, these findings suggest that relative VL dominance over the VM may be an important factor to consider when evaluating muscle coordination related to knee loading. However, ACL injuries typically occur during rapid, high-velocity movements that unfold within milliseconds [21], making direct assessment of the instantaneous force-sharing or power output of individual quadriceps muscles under injury-relevant conditions challenging. Ultrasound-based measures of muscle quality, including muscle echo intensity and shear modulus, have been proposed as laboratory-based surrogate indicators of neuromuscular performance. Even when assessed at rest, these measures have demonstrated associations with determinants requiring rapid force generation, such as joint power and rate of torque/power development [22–25]. This approach also enables muscle-specific assessment that may reflect functional capabilities. Therefore, evaluating the relative muscle quality characteristics of the VM and VL may provide a preliminary and physiologically meaningful surrogate indicator for exploring potential neuromuscular contributors to ACL loading.

Despite the potential importance of these indices, whether adolescent female soccer players exhibit specific H:Q and VM:VL profiles compared with recreationally active peers remains unclear. Characterizing normative profiles and exploring potential discrepancies in these indices within this population represent important preliminary steps toward establishing reference data for future injury-prevention research. Therefore, the purpose of this study was to investigate the muscular profiles of adolescent female soccer players, with particular emphasis on the isotonic H:Q ratio and VM:VL quality characteristics, while accounting for the potential effects of age and leg dominance. We hypothesized that: (1) adolescent female soccer players would exhibit lower isotonic H:Q ratios and lower relative VM:VL muscle quality ratios than recreationally active controls, and (2) the H:Q ratio derived from isotonic power would demonstrate a clear discrepancy from that derived from conventional isometric torque, reflecting the different physiological demands of dynamic versus static force production.

## Methods

### Participants

Participants (aged 12–17 years) were recruited from local junior and senior high schools and a professional soccer academy. The soccer group comprised elite players from the academy of a professional Japan Women’s Empowerment Professional Football League (WE League) club, representing highly trained athletes with consistent competitive backgrounds. The control group consisted of age-matched female students recruited primarily from local junior and senior high schools, with a small number additionally recruited during local sports-related events.

**Fig. 1:**
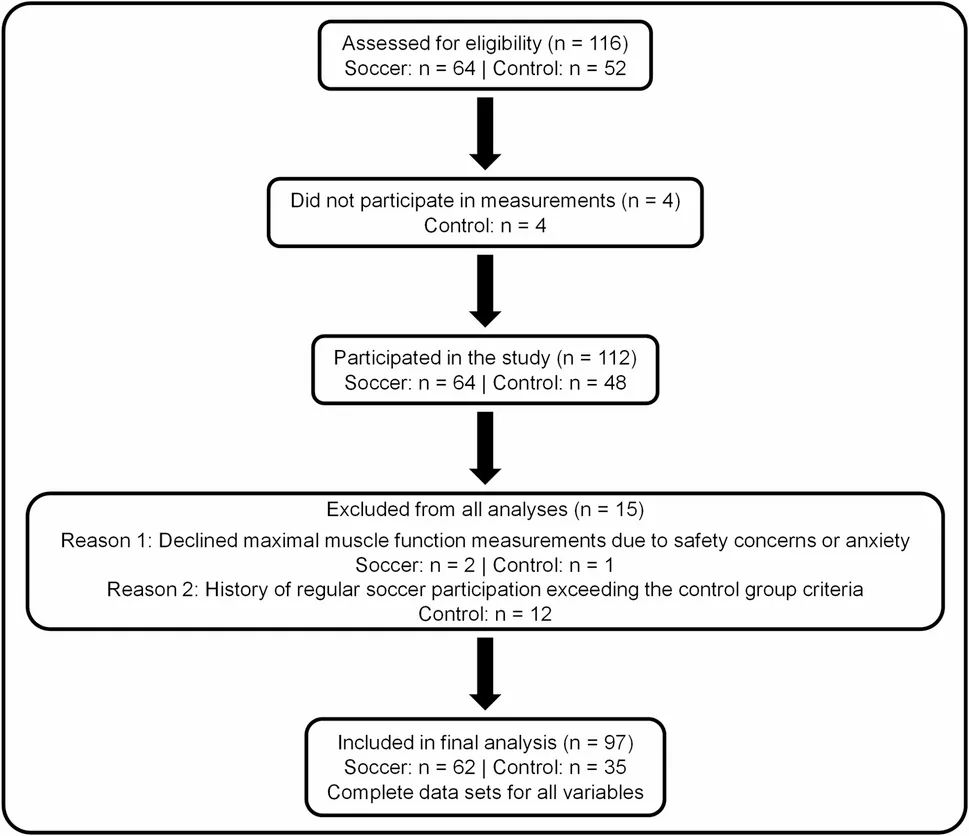
Flowchart of participant recruitment and selection. The final analysis included 97 participants (soccer: *n* = 62; control: *n* = 35) after exclusions for nonattendance (*n* = 4), refusal of maximal effort testing (*n* = 3), and regular soccer experience among control participants (*n* = 12)

Figure 1 shows the participant recruitment and selection flowchart. A total of 116 participants were initially assessed for eligibility, including 64 soccer players and 52 controls. Four control participants did not attend the measurement sessions. Accordingly, the remaining 112 participants attended the measurement sessions. However, 15 participants were subsequently excluded from all analyses: 3 (soccer group, *n* = 2; control group, *n* = 1) declined maximal muscle contraction testing owing to safety concerns or anxiety, and 12 controls were found to be engaged in organized soccer activities (defined as ≥ 3 sessions/week). The final sample consisted of 97 participants (soccer group, *n* = 62; control group, *n* = 35) with complete data for all study variables. No participants had a history of ACL injury or other major lower-limb musculoskeletal injuries requiring surgery or prolonged interruption of sports activities. 

The soccer group had a mean playing experience of 8 ± 3 years (mean ± standard deviation [SD]; range: 2–13 years). The academy training environment typically involved sessions 5–6 days per week with varied intensities. One day per week was allocated to an official or practice match, preceded by a low-intensity conditioning session the previous day. The remaining 3–4 days primarily consisted of moderate-intensity, skill-based sessions focusing on technical and shooting drills. Only one session per week was designated as high-intensity and involved opponent-focused, game-based drills. Formal resistance training was implemented only for high school–aged players at a frequency of one session per week and included fundamental lower-limb exercises such as squats and lunges targeting the knee extensors and flexors. The control group comprised 35 female adolescents who had not participated in organized soccer activities (defined as ≥ 3 sessions/week) for at least 1 year. Within this group, 16 participants reported either no prior history of regular exercise or no engagement in regular physical activity for at least 6 months prior to the commencement of the study. The remaining 19 participants had regularly engaged in various sports for at least 6 months leading up to the study; these activities included basketball (*n* = 8), badminton (*n* = 4), tennis or soft tennis (*n* = 3), and others such as track and field, table tennis, paddling, and dance. A small subset (*n* = 4) of the control group reported minimal prior exposure to soccer (defined as either monthly participation or having been away from regular soccer participation for > 5 years). Importantly, both the overall volume and intensity of physical activity in the control group were substantially lower than those of the elite soccer cohort, particularly with respect to sport-specific mechanical loading of the lower limbs. This heterogeneity reflected the available recruitment pool from local schools and was intentionally maintained to represent a general adolescent population with varied physical activity backgrounds and to facilitate comparison with the highly specialized, high-volume training demands of elite soccer. Furthermore, including recreationally active individuals ensured that the control group possessed the physical capacity required to reliably perform maximal-effort muscular assessments, while remaining distinct from the elite soccer players in terms of sport-specific mechanical loading.

This study was approved by the Institutional Review Board of the authors’ affiliated institution (No. 24 − 019/25 − 003) and conducted in accordance with the principles of the Declaration of Helsinki. The participants and their parents were informed of the study’s purpose and potential risks and provided written informed consent before participation.

### Experimental procedures

This cross-sectional study was conducted as part of a larger ongoing research project focusing on adolescent female soccer players. Consequently, a portion of the ultrasonography and muscle function data from a subset of participants (*n* = 70) overlapped with that reported in our previous study [26], which investigated muscular characteristics associated with hamstring strain injuries. In contrast, the present investigation focused on neuromuscular profiles relevant to ACL injury mechanisms by newly analyzing isotonic power-based H:Q ratios and the relative distribution of muscle quality between the VM and VL. These analyses provided a distinct functional perspective that was not explored in our previous work. Although the fundamental measurement setup, including the equipment and participant positioning, was consistent with our previous study [26], several parameters were newly derived and analyzed to address the specific objectives of the present investigation.

All measurements were performed bilaterally, with each leg tested separately. The dominant leg was defined as the leg used to kick a ball. In brief, isometric peak torque and isotonic peak power during maximal voluntary efforts were measured using a multi-mode dynamometer (CON-TREX MJ; Physiomed, Schnaittach, Germany). These data were recorded at 2 kHz using a PC application (LabChart v8.1.24, ADInstruments, Bella Vista, Australia) via an A/D converter (PowerLab16/35, ADInstruments). Additionally, muscle shear modulus and echo intensity were assessed at rest using a 45-mm linear transducer array (9L4 Transducer, 4–9 MHz, Siemens Medical Solutions, Malvern, PA, USA) connected to an ultrasound system (ACUSON S3000; Siemens Medical Solutions).

All evaluations for each participant were completed during a single testing session, with adequate rest periods provided between measurements to minimize fatigue. The order of ultrasound measurements and maximal-effort assessments, as well as the testing order of the legs, muscle groups, and movement directions, were randomized and counterbalanced across participants. Standardized verbal encouragement was provided throughout all maximal-effort assessments to ensure maximal participant effort.

### Muscle echo intensity and shear modulus measurements

At the beginning of the ultrasound measurement, thigh length was measured from the greater trochanter to the popliteal crease using a steel tape to the nearest 0.5 cm while participants stood upright. To standardize measurement sites, ink marks were placed at 80% and 50% of the thigh length (measured from the proximal end) for the assessment of the VM and VL, respectively. These sites were selected based on our previous observations indicating that the anatomical cross-sectional area of each muscle is expected to be large at these levels, thereby providing potentially representative values for each muscle belly. Following site marking, participants assumed a supine position on an examination bed. They were instructed to remain relaxed, with their ankles maintained in an approximately neutral position. To prevent internal or external hip rotation, the feet were positioned with the toes pointing upward. Ultrasound settings were optimized to visualize the muscles with appropriate penetration, and the bars for adjusting the brightness at each depth were aligned to the far right. Tissue harmonic imaging with a receiving frequency of 7-MHz was used, and the imaging gain and dynamic range were set at 0 and 70 dB, respectively, to ensure smooth muscle visualization. To identify the specific measurement sites, the examiner gently placed the linear transducer array transversely at 80% (VM) or 50% (VL) of thigh length. A water-soluble transmission gel was applied to the skin to ensure acoustic coupling. After identifying medial and lateral borders of each muscle, an ink mark was placed at the center of the muscle belly. For the assessment of muscle echo intensity, three cross-sectional B-mode images were acquired for each muscle. Between acquisitions, the transducer was completely removed and repositioned to ensure independence among trials. Muscle shear modulus was subsequently evaluated using shear-wave elastography. Longitudinal elastographic images were obtained using a color-coded scale ranging from blue (soft) to red (hard). To account for muscle anisotropy, the examiner adjusted the transducer orientation to be parallel to the muscle fascicles while observing the corresponding B-mode image. Image quality was monitored using a color-coded quality map ranging from green (good] to orange (poor). Images were considered acceptable only when yellow-to-orange pixels occupied less than 20% of the region of interest (ROI). This procedure was repeated until three high-quality elastographic images were acquired for each muscle. As with the echo intensity measurements, the transducer was repositioned before each acquisition. Throughout image collection, minimal probe pressure was applied to avoid artifacts resulting from tissue compression. All acquired images were exported in DICOM format to a personal computer for subsequent analysis.

Muscle echo intensity was analyzed using ImageJ software (National Institutes of Health, Bethesda, MD, USA) on the cross-sectional B-mode images. The ROI was defined as large as possible while carefully excluding surrounding fascia, and the mean gray-level within the ROI was calculated. For each muscle, the average value from the three images was used for further analysis. For muscle shear modulus analysis, longitudinal elastographic images were examined. ImageJ (National Institutes of Health, Bethesda, MD, USA) was first used to define the ROIs by maximizing their spatial extent while minimizing the inclusion of non-target tissues, such as subcutaneous adipose tissue, aponeuroses, and non-target muscles. The mean shear-wave propagation speed within each ROI was then calculated for each image using custom analysis software developed in MATLAB (MATLAB R2018a, MathWorks, Natick, MA, USA) [27]. The muscle shear modulus was calculated as the product of the muscle density (1,084 kg/m^3^) [28] and the square of the average shear-wave propagation speed obtained from the three images [29]. Finally, the VM:VL ratio for both echo intensity and shear modulus was calculated by dividing the VM value by the corresponding VL value to evaluate the relative muscle quality characteristics between the two muscles.

To assess the intra-rater reliability of the measurements, the coefficient of variation (CV) and intraclass correlation coefficient (ICC) were calculated from the three images acquired for all 194 legs (97 participants × 2 legs). The CV and ICC (1,3) were 3.8% ± 2.6% and 0.956 (*P* < 0.001) for VM echo intensity, 3.2% ± 2.5% and 0.965 (*P* < 0.001) for VL echo intensity, 3.2% ± 2.2% and 0.941 (*P* < 0.001) for VM shear-wave propagation speed, and 4.5% ± 2.8% and 0.930 (*P* < 0.001) for VL shear-wave propagation speed, respectively.

Additionally, the day-to-day reliability of muscle echo intensity and shear-wave propagation speed measurements was evaluated in both legs of a separate group of six female participants in their 20s (*n* = 12 limbs) over two testing sessions conducted on separate days. These participants were recruited specifically for the reliability assessment and were not included in the main study cohort. The resulting CV and ICC (1,1) values were 6.2% ± 3.2% and 0.673 (*P* = 0.005) for VM echo intensity, 2.7% ± 1.8% and 0.814 (*P* < 0.001) for VL echo intensity, 5.8% ± 5.1% and 0.603 (*P* = 0.012) for VM shear-wave propagation speed, and 3.2% ± 2.0% and 0.882 (*P* < 0.001) for VL shear-wave propagation speed, respectively.

### Isometric peak torque and isotonic peak power measurements

For each muscle group and leg, the measurement of isometric peak torque consistently preceded that of isotonic peak power. This sequence was necessary to determine individual resistances for the subsequent isotonic assessments, which were set relative to the isometric peak torque values.

Participants were seated in the dynamometer with the hip joint fixed at 80° of flexion. For isometric assessments, the knee joint was positioned at 30° flexion for the knee flexors and 70° for the knee extensors (anatomical position = 0°). To minimize extraneous movement, the pelvis and torso were firmly secured to the seat using non-elastic straps and a seat belt. The axis of rotation of the dynamometer was carefully aligned with the center of the knee joint. A lever-arm ankle adapter was secured slightly proximal to the lateral malleolus. Following a standardized warm-up consisting of 2–3 submaximal contractions, participants performed two 3-s isometric maximal voluntary contractions for each muscle group, separated by a 1-min rest period. If the difference between the two peak torque values exceeded 10% of the higher value, additional trials were performed until the discrepancy between the two highest values was within 10%. During offline analysis, the isometric torque signals were processed using a 500-Hz low-pass filter, and the highest peak torque recorded across all successful trials was adopted. Furthermore, the isometric H:Q ratio was calculated as the ratio of knee flexor peak torque to knee extensor peak torque.

Subsequently, following a warm-up of 2–3 submaximal contractions, participants performed five consecutive maximal-effort isotonic contractions for each muscle group. The resistance was set to 10% of the pre-measured isometric peak torque to reflect the low-load, high-velocity conditions typical of athletic maneuvers, such as the swing phase of kicking, where the primary load is the inertia of the lower limb. The range of motion was from 30° to 110° for the knee flexors and from 110° to 30° for the knee extensors. Once the lever arm reached the end of the range, it automatically returned to the starting position over a 1-s period while the participants remained fully relaxed. During offline analysis, the torque and angular velocity signals were processed using a 20-Hz low-pass filter. Power output was calculated as the product of joint torque and angular velocity, and the highest peak power value recorded across the five trials was used for further analysis. Additionally, the isotonic H:Q ratio was calculated as the ratio of the knee flexor peak power to the knee extensor peak power.

### Statistical analyses

To account for developmental heterogeneity, chronological age in months was recorded for all participants. Within the soccer group, subgroups were defined using cluster analysis (Ward’s method) based on chronological age. This analysis identified two distinct groups: an “older soccer” group (*n* = 36; ≥ 162 months) and a “younger soccer” group (*n* = 26; < 162 months). Participants in the control group were subsequently assigned to corresponding age categories using the same threshold, resulting in an “older control” group (*n* = 24; ≥ 162 months) and a “younger control” group (*n* = 11; < 162 months). These categorical groups, together with leg dominance, were used to facilitate comparisons between soccer players and recreationally active controls while statistically accounting for potential developmental and bilateral variations. The minimum required sample size was pre-determined to be 48 participants based on the requirements for three-way analysis of variance (ANOVA) using G*Power software (version 3.1.9.6; Kiel University, Kiel, Germany) with the following parameters: Test family = F tests; Statistical test = ANOVA: Repeated measures, within-between interaction; Effect size: f = 0.25 (medium) [30]; α err prob = 0.05; Power (1 − β err prob) = 0.80; Number of groups = 4; Number of measurements = 2; Corr among rep measures = 0.50; and Nonsphericity correction e = 1. The final sample size of 97 participants exceeded this threshold, ensuring adequate statistical power for all muscle-related variables analyzed using the same ANOVA design. Table 1 summarizes age (both in years and months), height, weight, and body mass index of each group, as well as soccer career (in years) for the soccer groups.

**Table 1:** Anthropometric characteristics and soccer experience of the participants across age and experience groups

Parameter	Group	Mean	±	SD	ANOVA effects	F	t	*P*
Age (years)	older soccer	15	±	1	interaction			
	younger soccer	12	±	0.3	age × experience	*F*[1,93] = 2.711		0.103
	older control	15	±	1	main effect			
	younger control	12	±	1	Age	*F*[1,93] = 171.343		< 0.001
					experience	*F*[1,93] = 0.424		0.517
Age (months)	older soccer	183	±	13	interaction			
	younger soccer	150	±	4	age × experience	*F*[1,93] = 5.195		0.025
	older control	178	±	12	simple main effect			
	younger control	154	±	3	age: soccer	*F*[1,93] = 192.193		< 0.001
					age: control	*F*[1,93] = 49.115		< 0.001
					experience: older	*F*[1,93] = 4.142		0.045
					experience: younger	*F*[1,93] = 1.781		0.185
Height (cm)	older soccer	161.3	±	5.3	interaction			
	younger soccer	156.6	±	4.0	age × experience	*F*[1,93] = 0.132		0.717
	older control	158.1	±	5.4	main effect			
	younger control	154.3	±	6.2	Age	*F*[1,93] = 13.661		< 0.001
					experience	*F*[1,93] = 5.666		0.019
Mass (kg)	older soccer	53.0	±	5.6	interaction			
	younger soccer	45.5	±	3.8	age × experience	*F*[1,93] < 0.001		0.992
	older control	49.4	±	6.3	main effect			
	younger control	41.9	±	5.7	Age	*F*[1,93] = 39.714		< 0.001
					experience	*F*[1,93] = 9.146		0.003
Body mass index (kg/m^2^)	older soccer	20.4	±	1.8	interaction			
	younger soccer	18.5	±	1.1	age × experience	*F*[1,93] = 0.205		0.652
	older control	19.8	±	2.3	main effect			
	younger control	17.5	±	1.8	Age	*F*[1,93] = 24.870		< 0.001
					experience	*F*[1,93] = 3.846		0.053
Soccer career (years)	older soccer	9	±	2			*t*(60) = 4.47	< 0.001
	younger soccer	7	±	2				

Participants were categorized into four subgroups based on soccer experience (elite soccer academy members vs. recreationally active controls) and chronological age (older: ≥ 162 months vs. younger: < 162 months). Differences in anthropometric parameters were evaluated using a two-way analysis of variance (ANOVA), with age and experience included as between-subject factors. For soccer career, an independent *t*-test was performed between the older and younger soccer groups. *F* and *t* values with degrees of freedom, and corresponding *P*-values, are presented to indicate statistical significance

*SD *standard deviation

The primary outcomes of this study were the isotonic H:Q ratio, isometric H:Q ratio, and relative VM:VL muscle quality ratio, which directly addressed the study hypotheses. Individual joint performance data and muscle-specific characteristics used to calculate these indices were considered secondary or exploratory outcomes.

Differences in all dependent variables were evaluated using a three-way mixed-design ANOVA, with leg (dominant vs. non-dominant) as the within-subject factor, and age (older vs. younger) and experience (soccer vs. control) as the between-subject factors. This analytical approach was selected to account for the potential confounding effects of age and leg dominance on neuromuscular characteristics. In the event of a significant three-way interaction, the analysis was decomposed into age × experience simple interactions for each leg. Significant simple interactions were followed by simple-simple main effects analyses with Bonferroni adjustments to identify specific group differences. When neither three-way nor two-way interactions were significant, the main effects of experience, age, and leg were interpreted directly.

Before conducting the primary analyses, normality of the data distribution was assessed using the Shapiro–Wilk test. All variables, except the knee flexor and extensor peak power, were log-transformed to satisfy the assumptions of ANOVA.

All statistical analyses were performed using SPSS (version 28.0; IBM, Armonk, NY, USA) and Microsoft Excel (2016; Microsoft, Redmond, WA, USA). For ease of interpretation, descriptive data presented in the text and figures are reported as raw values (mean ± SD or range [minimum–maximum]), regardless of whether log transformation was applied. Statistical significance was set at *P* < 0.05. Effect sizes in the three-way ANOVA were expressed as generalized eta squared (*η*_G_^2^), calculated according to Bakeman [31] based on the formulation proposed by Olejnik and Algina [32]. Effect sizes were interpreted using Cohen’s conventional benchmarks [30]: trivial (< 0.010), small (0.010–0.059), medium (0.060–0.139), and large (≥ 0.140).

### Declaration of AI use in the writing process

During the preparation of this manuscript, the authors used Gemini (Google) during the initial drafting phase to improve the English language, readability, and sentence structure prior to final professional editing. Following the use of this tool, the authors reviewed and edited the content as needed and take full responsibility for the content of the publication.

## Results

The complete statistical outcomes, including *F*-, *P*-, and *η*_G_^2^- values, for knee flexor and extensor peak torque and power, isometric and isotonic H:Q ratios, muscle echo intensity and shear modulus of the VM and VL, and the VM:VL ratios of muscle echo intensity and shear modulus are provided in Additional Files 1–4.

**Fig. 2:**
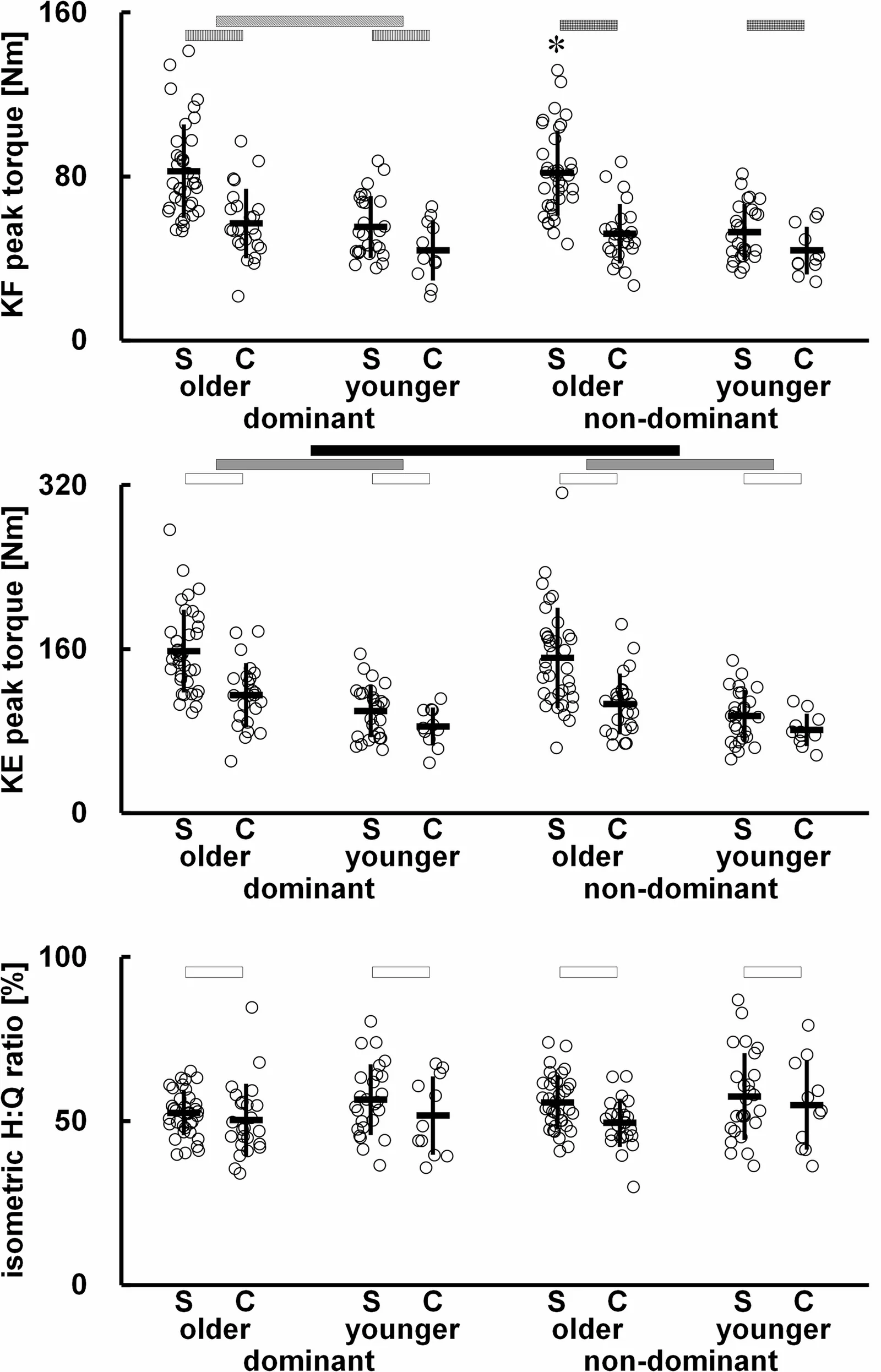
Isometric knee flexor (KF) and extensor (KE) peak torques and hamstring-to-quadriceps (H:Q) ratio. Bold horizontal lines represent means; thin vertical lines represent standard deviations; circles indicate individual raw data points. Statistical annotations above each plot are defined as follows: - Asterisk (*): Significant simple-simple main effect of age within the soccer group for the non-dominant leg (older > younger). - Horizontal lines with a lattice pattern: Significant simple-simple main effect of experience for the non-dominant leg in both the older and younger groups. - Horizontal lines with diagonal stripes (top-left to bottom-right): Significant simple main effect of age for the dominant leg. - Horizontal lines with vertical stripes: Significant simple main effect of experience for the dominant leg. - Black horizontal lines: Significant main effect of leg. - Gray horizontal lines: Significant main effect of age. - White horizontal lines: Significant main effect of experience. S, soccer group; C, control group. Older cohort: ≥162 months (~13.5 years), *n* = 36 (S) and *n* = 24 (C); Younger cohort: <162 months, *n* = 26 (S) and *n* = 11 (C)

Knee flexor and extensor peak torque, together with the isometric H:Q ratio, are presented in Fig. 2. For knee flexor peak torque, a significant leg × age × experience interaction was observed (*P* = 0.044). In the dominant leg, significant simple main effects of age (older > younger) and experience (soccer > control) were observed (*P* < 0.001), whereas the age × experience simple interaction was not significant. In the non-dominant leg, a significant age × experience simple interaction was observed (*P* = 0.021). Subsequent simple-simple main effect analyses revealed that: (1) soccer experience had a significant effect regardless of age (*P* ≤ 0.049); (2) age had a significant effect within the soccer group (*P* < 0.001); and (3) no significant age effect was observed within the control group. Regarding knee extensor peak torque, significant main effects of leg (dominant > non-dominant), age (older > younger), and experience (soccer > control) were observed (*P* ≤ 0.002), with no significant interactions. For the isometric H:Q ratio, only a significant main effect of experience was identified (soccer: 55.3% ± 9.8%, control: 51.0% ± 10.4%; mean difference in log-transformed values: 0.079 [back-transformed ratio: 1.082], 95% confidence interval [CI]: 0.008 to 0.151 [back-transformed 95% CI: 1.008 to 1.163]; *P* = 0.029; *η*_G_^2^ = 0.038; pooled across all age groups and legs). No other significant main effects or interactions were observed.

**Fig. 3:**
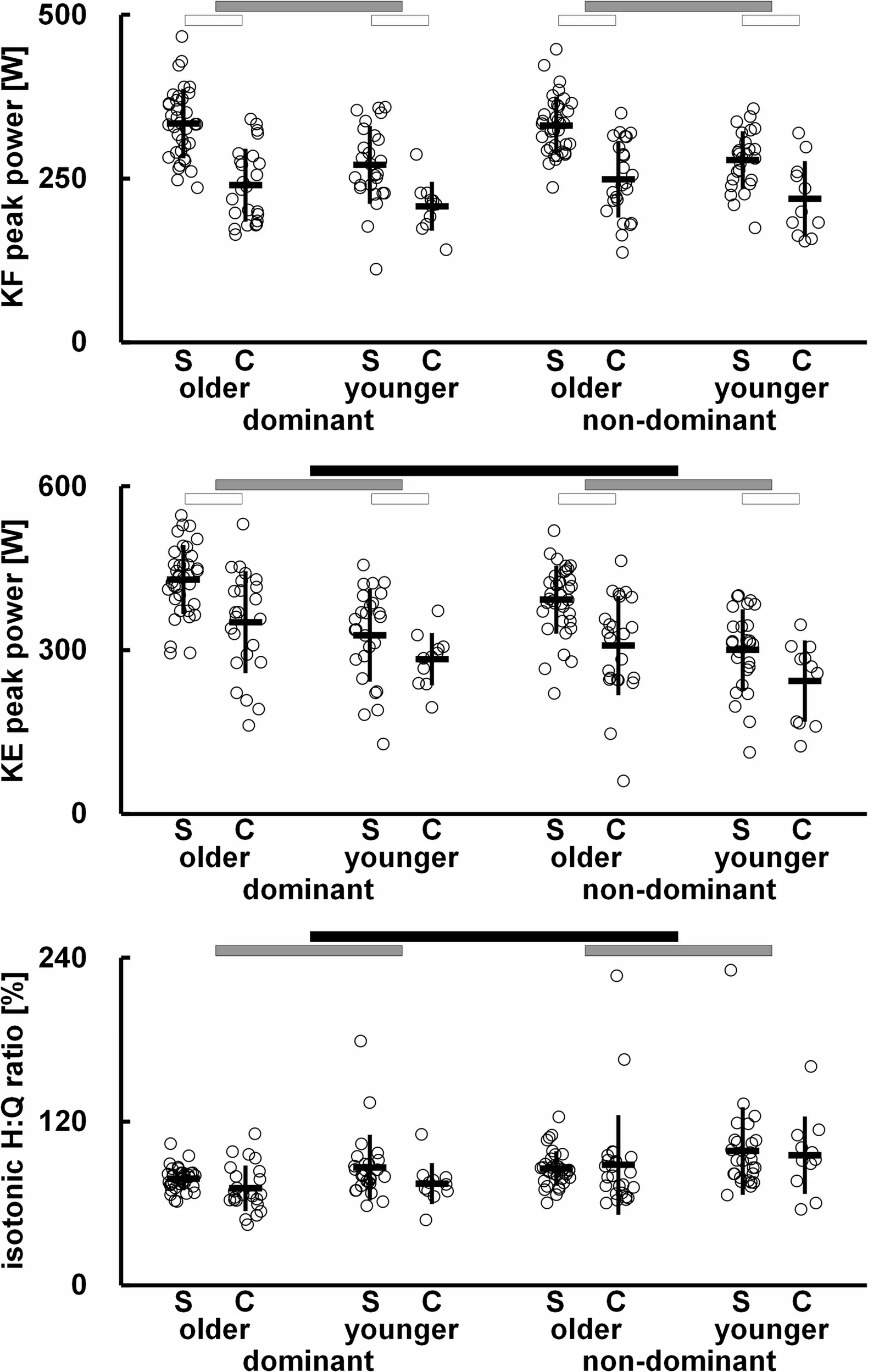
Isotonic knee flexor (KF) and extensor (KE) peak power and hamstring-to-quadriceps (H:Q) ratio. Bold horizontal lines represent means; thin vertical lines represent standard deviations; circles indicate individual raw data points. Lines above plots indicate significant main effects: leg (black), age (gray), and experience (white). S, soccer group; C, control group. Older cohort: ≥162 months (~13.5 years), *n* = 36 (S) and *n* = 24 (C); Younger cohort: <162 months, *n* = 26 (S) and *n* = 11 (C)

Figure 3 presents knee flexor and extensor peak power, as well as the isotonic H:Q ratio. For knee flexor peak power, significant main effects of age (older > younger) and experience (soccer > control) were observed (*P* < 0.001), while knee extensor peak power showed significant main effects for leg, age, and experience (*P* < 0.001; dominant > non-dominant, older > younger, and soccer > control, respectively). No significant two-way or three-way interactions were observed for either peak power measure. Regarding the isotonic H:Q ratio, significant main effects of leg (dominant < non-dominant) and age (older < younger) were observed (*P* ≤ 0.040), with no significant interactions or main effect of experience (soccer: 86.3% ± 21.1%, control: 81.4% ± 27.9%; mean difference in log-transformed values: 0.074 [back-transformed ratio: 1.077], 95% CI: −0.006 to 0.155 [back-transformed 95% CI: 0.994 to 1.168]; *P* = 0.069; *η*_G_^2^ = 0.024; pooled across all age groups and legs).

**Fig. 4:**
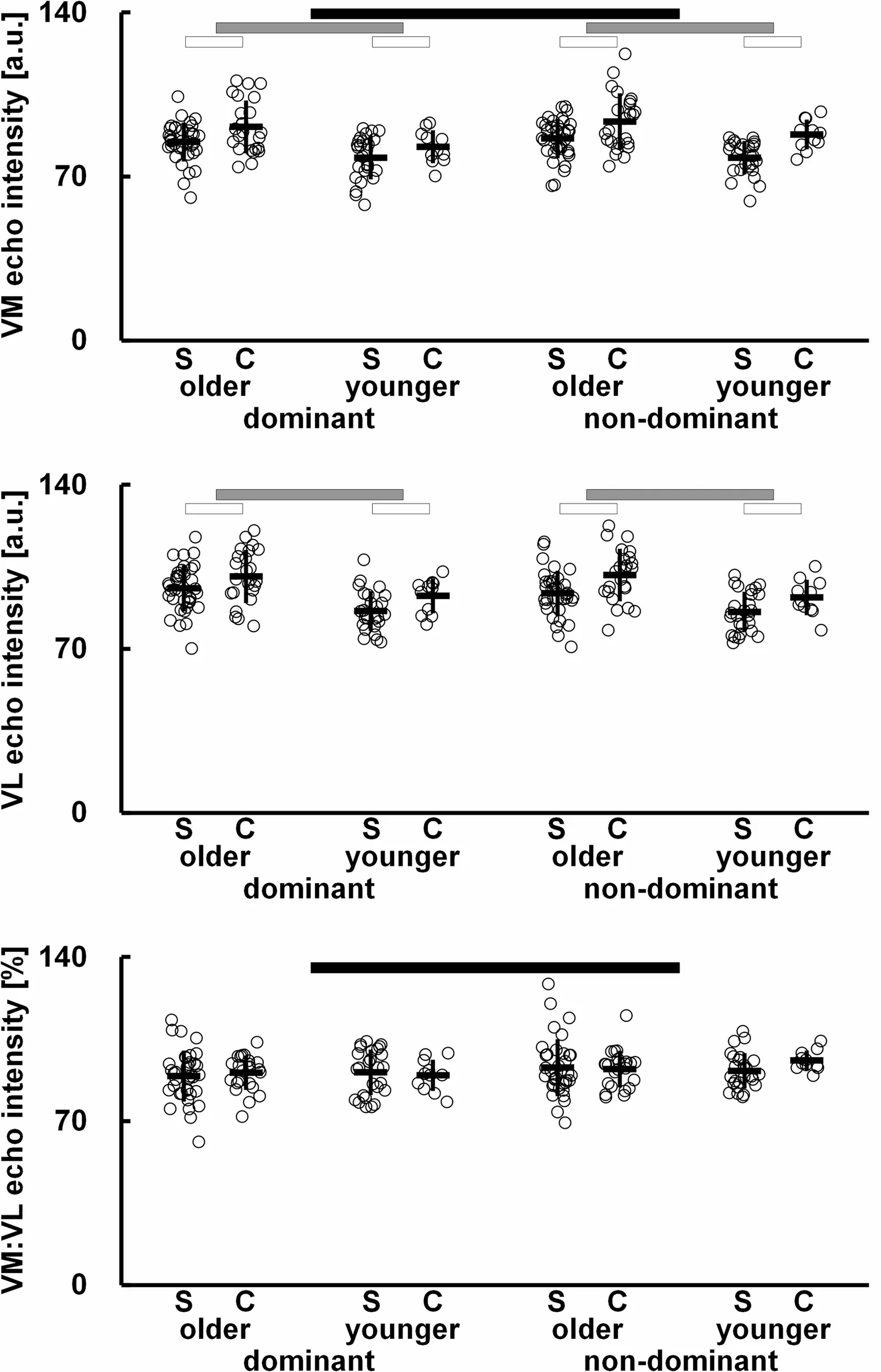
Echo intensity of vastus medialis (VM) and vastus lateralis (VL), and the VM:VL ratio. Bold horizontal lines represent means; thin vertical lines represent standard deviations; circles indicate individual raw data points. Lines above plots indicate significant main effects: leg (black), age (gray), and experience (white). S, soccer group; C, control group. Older cohort: ≥162 months (~13.5 years), *n* = 36 (S) and *n* = 24 (C); Younger cohort: <162 months, *n* = 26 (S) and *n* = 11 (C)

Figure 4 presents the echo intensity of the VM and VL, and the VM:VL echo intensity ratio. No significant two-way or three-way interactions were observed for either muscle. Significant main effects for all factors were observed for VM echo intensity (*P* ≤ 0.011; dominant < non-dominant, older > younger, and soccer < control, respectively), whereas only the significant main effects of age (older > younger) and experience (soccer < control) were observed for the VL echo intensity (*P* ≤ 0.002). Regarding the VM:VL ratio of echo intensity, only a significant main effect of leg was observed (*P* = 0.016; dominant < non-dominant), with no significant interactions or main effects of age and experience (soccer: 91.0% ± 10.4%, control: 91.8% ± 7.2%; mean difference in log-transformed values: −0.014 [back-transformed ratio: 0.986], 95% CI: −0.051 to 0.022 [back-transformed 95% CI: 0.950 to 1.022]; *P* = 0.441; *η*_G_^2^ = 0.004; pooled across all age groups and legs).

**Fig. 5:**
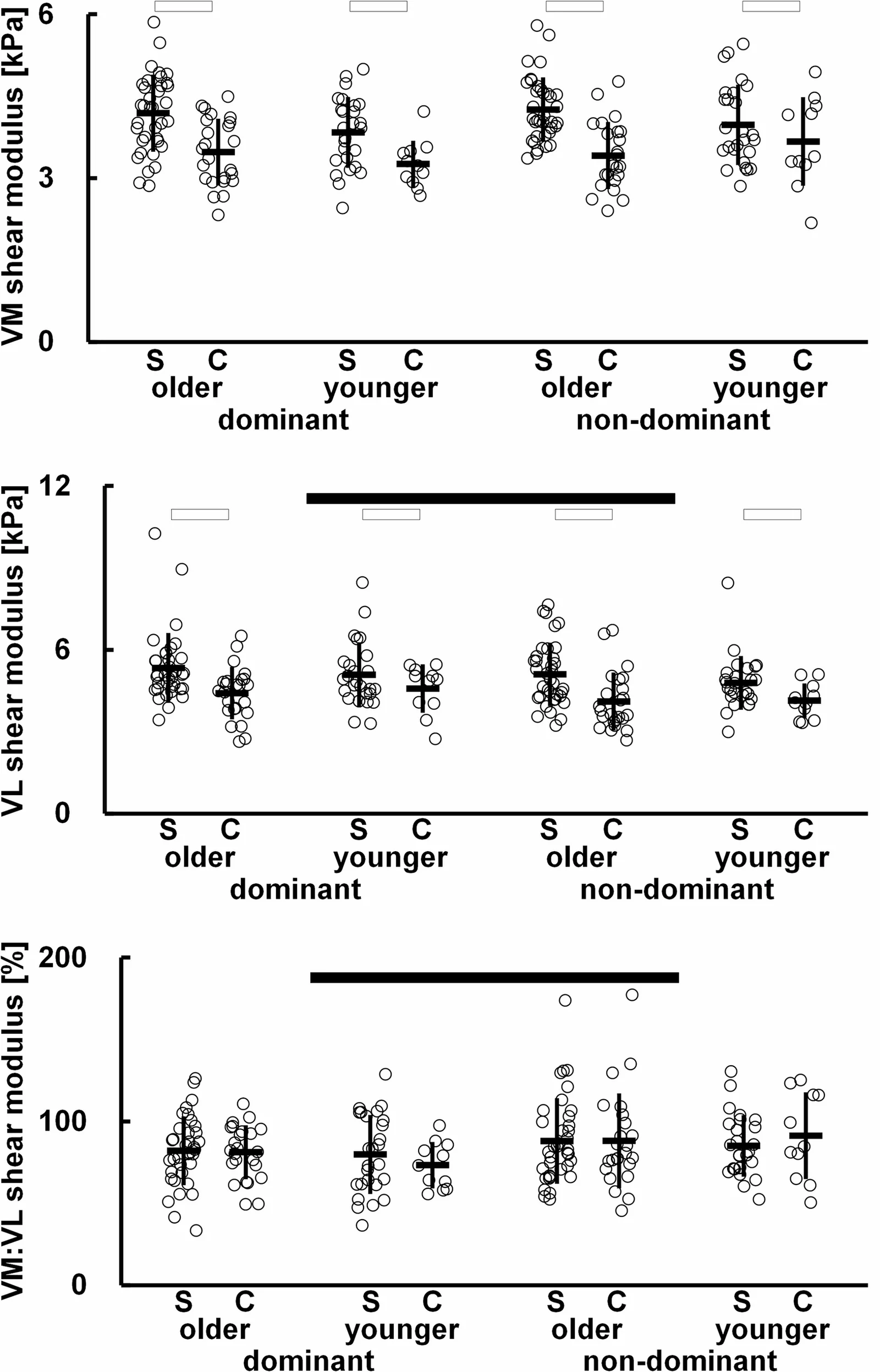
Shear modulus of vastus medialis (VM) and vastus lateralis (VL), and the VM:VL ratio. Bold horizontal lines represent means; thin vertical lines represent standard deviations; circles indicate individual raw data points. Lines above plots indicate significant main effects: leg (black) and experience (white). S, soccer group; C, control group. Older cohort: ≥162 months (~13.5 years), *n* = 36 (S) and *n* = 24 (C); Younger cohort: <162 months, *n* = 26 (S) and *n* = 11 (C)

The VM and VL shear moduli, together with the VM:VL shear modulus ratio, are presented in Fig. 5. For both muscles, no significant three-way or two-way interactions and no main effect of age were observed. For VM shear modulus, only a significant main effect of experience was observed (*P* < 0.001), with higher values in soccer players than in controls. For VL shear modulus, significant main effects of leg (*P* = 0.004; dominant > non-dominant) and experience (*P* < 0.001; soccer > control) were observed. Regarding the VM:VL shear modulus ratio, only a significant main effect of leg was observed (*P* = 0.001; dominant < non-dominant), with no other significant interactions or main effects of age and experience (soccer: 84.0% ± 22.8%, control: 83.9% ± 23.2%; mean difference in log-transformed values: 0.002 [back-transformed ratio: 1.002], 95% CI: −0.102 to 0.106 [back-transformed 95% CI: 0.903 to 1.112]; *P* = 0.975; *η*_G_^2^ < 0.001; pooled across all age groups and legs).

## Discussion

The main findings of this study were as follows: (1) although the isometric H:Q ratio was significantly higher in the soccer group than in the control group, no significant inter-group difference was observed in the isotonic H:Q ratio; and (2) no significant between-group differences were observed in either the VM:VL echo intensity ratio or the VM:VL shear modulus ratio. Thus, the results did not support most aspects of our first hypothesis but supported our second hypothesis. In the following discussion, we primarily focus on the comparisons between the soccer and control groups to characterize the neuromuscular profiles of adolescent female soccer players, as the effects of age and leg dominance were not consistent across the measured parameters.

Importantly, because the present findings were derived from a cross-sectional study, they describe specific neuromuscular profiles rather than causative factors for injury. Therefore, although several significant differences were identified, the results do not establish causal relationships between the identified characteristics and the occurrence of ACL injury. To further strengthen the clinical implications of the findings, future longitudinal studies are needed to examine how these neuromuscular profiles evolve within the individuals over time. Furthermore, prospective studies tracking ACL injury incidence are required to determine the clinical relevance of the laboratory-based surrogate measures identified in this study.

For the knee extensors, both isometric peak torque (Fig. 2) and isotonic peak power (Fig. 3) were significantly greater in the soccer group than in the control group, regardless of age or leg dominance. Similarly, for the knee flexors, although the influence of age or leg dominance varied slightly between the contraction modes (isometric vs. isotonic), the soccer group consistently demonstrated greater values than the control group across all age and leg dominance subgroups (Figs. 2 and 3). Kicking is characterized primarily by rapid knee extension [33] although rapid knee flexion also plays an important role in optimizing performance [34]. Furthermore, studies involving male soccer players have shown that elite athletes possess higher isokinetic torques than their sub-elite counterparts [35], and that elite players exhibit greater hamstring strength than amateur players, suggesting that hamstring strength is an important characteristic associated with soccer performance [36]. Thus, the superior muscle function in our soccer group reflects their competitive profiles, including the effects of structured physical preparation.

A distinct discrepancy emerged between contraction modes when the H:Q ratio was considered. The isometric H:Q ratio showed only a significant inter-group difference (soccer > control), with no influence of age or leg dominance (Fig. 2). In contrast, the isotonic H:Q ratio was influenced by age (older < younger) and leg dominance (dominant < non-dominant), whereas no significant difference was observed between the soccer and control groups (Fig. 3). The absence of an age-related effect on the isometric H:Q ratio is consistent with previous findings reporting that conventional and functional ratios were similar across all age groups, with the exception of slightly higher values in the dominant limb of the under-13 compared with the under-15 age groups [37]. Because actual bodily movements involve joint motion under dynamically changing angular velocities, the isotonic H:Q ratio results may provide complementary perspective that better reflects dynamic muscle performance during movement. However, the present findings demonstrate differences between contraction modes rather than establishing the clinical superiority or predictive validity of the isotonic measurements relative to isometric measurements. Furthermore, because the isotonic H:Q ratio did not differ significantly between soccer players and age-matched controls, the lower values observed in older participants may reflect a general developmental profile associated with female adolescence rather than a characteristic unique to soccer participation. In this regard, the potential for Type II error could be considered given the *p*-value for the group effect on the isotonic H:Q ratio (*P* = 0.069). This trend might have been influenced by the heterogeneity of the control group, potentially limiting the internal validity of this comparison; however, the small effect size (*η*_G_^2^ = 0.024) suggests that any inter-group difference may be relatively limited.

Regardless of the muscle (VM or VL) or leg (dominant or non-dominant), the soccer group exhibited significantly lower echo intensity (Fig. 4) and significantly higher shear modulus (Fig. 5) than the control group. Although muscle echo intensity has been established as a useful tool for capturing age-related changes in muscle quality, its validity in healthy young populations has remained relatively limited [38]. Nevertheless, Cruz-Montecinos et al. [39] demonstrated that muscle echo intensity can be used as an indicator of muscle quality reflecting intra-muscular fat and connective tissue in a similar age group to our participants. Furthermore, Terzis et al. [40] recently reported a positive correlation between muscle echo intensity and the proportion of Type I fibers in young females, suggesting that lower echo intensity may reflect a greater proportion of Type II (fast-twitch) fibers. Therefore, our findings tentatively suggest that adolescent female soccer players are characterized not only by fewer non-contractile elements but also by a fiber type composition potentially more favorable for rapid force generation compared to the age-matched control group. Interestingly, our finding that the older participants showed higher echo intensity than the younger participants contrasts with the findings of García-Alonso et al. [41] who reported a decrease in echo intensity with age in a younger cohort (4.0–10.9 years). Given the limited data available in healthy young populations [38], further investigation is required to clarify age-related changes in muscle echo intensity across developmental stages. Evidence regarding differences in muscle shear modulus between athletes and non-athletes remains inconsistent. Previous studies have reported that athletes’ muscles are softer [42], similar in stiffness [42–44], or stiffer [43, 44] than those of controls. Consequently, direct comparison of our findings with the existing literature is challenging. Nevertheless, the present data provide a novel reference for muscle quality profiles in adolescent female soccer players during a critical developmental period.

Considering these results collectively, we found no significant differences in the VM:VL ratios for either muscle echo intensity (Fig. 4) or shear modulus (Fig. 5) between the soccer and control groups. This indicates that, from the perspective of relative VM:VL characteristics, adolescent female soccer players do not exhibit statistically distinct muscular profiles compared with their age-matched peers. With respect to measurement precision, day-to-day reliability was acceptable for VM-related variables (CV: 6.2% for echo intensity and 5.8% for shear-wave propagation speed), although the corresponding ICCs (0.603–0.673) were lower than those for VL-related variables (0.814–0.882). To determine whether these region-specific differences in reliability may have influenced the ratio-based outcomes, we additionally analyzed the day-to-day reliability of the calculated ratios using the reliability cohort (*n* = 12 limbs). The resulting CV and ICC (1,1) values were 5.4% ± 3.6% and 0.685 (*P* = 0.004) for the echo intensity ratio, and 13.9% ± 10.6% and 0.721 (*P* = 0.002) for the shear modulus ratio, respectively. Although the CV for the shear modulus ratio was relatively high because of the mathematical derivation from square-transformed speed data, the overall reliability metrics suggest that examiner-related technical variability is unlikely to have substantially affected the primary null findings. However, acceptable reliability does not necessarily establish the construct validity of these VM:VL ratios. These resting, ultrasound-derived measures do not necessarily reflect dynamic quadriceps coordination, force-sharing, or neuromuscular control during actual athletic maneuvers. Therefore, the absence of significant between-group differences should not be interpreted as evidence that neither group exhibits suboptimal neuromuscular patterns; both populations may remain in a developmental stage characterized by vulnerability to impaired joint stability. Moreover, because these findings are based on group-level averages, individual variations must be considered. Some participants may still possess muscle quality profiles associated with asymmetries that have been linked to ACL loading mechanisms. Although the present findings do not establish injury risk, characterization of neuromuscular profiles in youth soccer players may provide valuable reference data for future injury-prevention research. In this context, structured programs such as FIFA 11 + Kids have been shown to substantially reduce injury incidence among young soccer players [45].

This study has several limitations that should be considered when interpreting the findings. Although chronological age in years was matched between groups, the older soccer group had a significantly higher age in months than the corresponding control group, and the soccer group was generally taller and heavier. Furthermore, the absence of direct indicators of biological maturation precludes exclusion of the potential confounding influence of advanced maturation on the results. Another important limitation relates to training background. Structured resistance training was implemented only among the high school–aged soccer players, whereas neither the younger soccer players nor the control group had such exposure. Consequently, differences in training history within the soccer cohort may have contributed to the observed age- and group-related differences in muscular characteristics. Additionally, the control group comprised recreationally active individuals with heterogeneous training backgrounds, which may have increased variability and attenuated potential between-group differences. Regarding testing conditions, measurements for many soccer players were conducted the day after a match or intense training session. As a result, residual fatigue or muscle swelling may have introduced systematic bias into both absolute and ratio-based outcomes of muscle quality and function. Moreover, because the isometric test was methodologically required to establish the load used during the subsequent isotonic test, a fixed testing order was unavoidable. Consequently, potential order effects, including neuromuscular potentiation or residual fatigue induced by maximal isometric contractions, cannot be completely excluded when interpreting the isotonic power outcomes. Finally, isotonic power was evaluated only under a low-load condition (10% of isometric peak torque). Therefore, the findings primarily reflect velocity-dominant muscle performance and may not be generalizable to situations involving high external loads and strong ground reaction forces, where ACL injuries typically occur. In addition, the VM:VL ratios were derived solely from static, resting-state ultrasound measurements. As these variables do not reflect dynamic synergistic activation or force-sharing during multi-joint movements, direct inferences regarding dynamic joint stabilization during athletic maneuvers should be made with caution.

## Conclusions

This cross-sectional study investigated the muscle characteristics of adolescent female soccer players, with a specific focus on the H:Q torque and power ratios and relative VM:VL muscle quality characteristics. The H:Q ratio calculated from isotonic power did not correspond to that derived from conventional isometric torque. Furthermore, no significant differences were observed between the soccer and control groups in the isotonic H:Q ratio or the VM:VL ratios of echo intensity and shear modulus. These findings suggest that adolescent female soccer players do not exhibit statistically detectable differences in these specific muscular profiles compared with age-matched controls within the scope of the parameters assessed in this study. These multi-faceted muscular profiles may serve as preliminary, laboratory-based surrogate reference data for monitoring neuromuscular characteristics in adolescent female athletes. Given the inconsistent effects of age and leg dominance across the measured parameters, a multi-faceted approach to muscular profile assessment appears warranted. These findings may also provide baseline reference data for future longitudinal or interventional studies aimed at clarifying the relationship between these muscle traits and actual injury incidence.

## Supplementary Information

Supplementary Material 1.

Supplementary Material 2.

Supplementary Material 3.

Supplementary Material 4.

## Data Availability

The datasets generated during and/or analyzed during the current study are available from the corresponding author on a reasonable request.

## Abbreviations

ACL: Anterior cruciate ligament
ANOVA: Analysis of variance
CI: Confidence interval
CV: Coefficient of variation
H:Q: Hamstring-to-quadriceps
ICC: Intraclass correlation coefficient
ROI: Region of interest
SD: Standard deviation
VL: Vastus lateralis
VM: Vastus medialis
WE League: Japan Women’s Empowerment Professional Football League

## References

[1] Mancino F, Kayani B, Gabr A, Fontalis A, Plastow R, Haddad FS. Anterior cruciate ligament injuries in female athletes: risk factors and strategies for prevention. Bone Jt Open. 2024;5:94–100. 10.1302/2633-1462.52.bjo-2023-0166. 38310925 PMC10838619

[2] Dewig DR, Boltz AJ, Moffit RE, Rao N, Collins CL, Chandran A. Epidemiology of anterior cruciate ligament tears in National Collegiate Athletic Association athletes: 2014/2015–2018/2019. Med Sci Sports Exerc. 2024;56:29–36. 10.1249/MSS.0000000000003281. 37616175

[3] Childers J, Eng E, Lack B, et al. Reported anterior cruciate ligament injury incidence in adolescent athletes is greatest in female soccer players and athletes participating in club sports: A systematic review and meta-analysis. Arthroscopy. 2025;41:774–84.e2. 10.1016/j.arthro.2024.03.050. 38692337

[4] Manojlovic M, Ninkovic S, Matic R, et al. Return to play and performance after anterior cruciate ligament reconstruction in soccer players: a systematic review of recent evidence. Sports Med. 2024;54:2097–108. 10.1007/s40279-024-02035-y. 38710914 PMC11329701

[5] Mazza D, Viglietta E, Monaco E, et al. Impact of anterior cruciate ligament injury on European professional soccer players. Orthop J Sports Med. 2022;10:23259671221076865. 10.1177/23259671221076865. 35224121 PMC8873562

[6] Fältström A, Kvist J, Hägglund M. High risk of new knee injuries in female soccer players after primary anterior cruciate ligament reconstruction at 5- to 10-year follow-up. Am J Sports Med. 2021;49:3479–87. 10.1177/03635465211044458. 34623936

[7] Kellis E, Sahinis C, Baltzopoulos V. Is hamstrings-to-quadriceps torque ratio useful for predicting anterior cruciate ligament and hamstring injuries? A systematic and critical review. J Sport Health Sci. 2023;12:343–58. 10.1016/j.jshs.2022.01.002. 35065297 PMC10199143

[8] Ruas CV, Pinto RS, Haff GG, Lima CD, Pinto MD, Brown LE. Alternative methods of determining hamstrings-to-quadriceps ratios: a comprehensive review. Sports Med Open. 2019;5:11. 10.1186/s40798-019-0185-0. 30911856 PMC6434009

[9] Hohmann E, Tetsworth K, Glatt V. The hamstring/quadriceps ratio is an indicator of function in ACL-deficient, but not in ACL-reconstructed knees. Arch Orthop Trauma Surg. 2019;139:91–8. 10.1007/s00402-018-3000-3. 30062456

[10] Baumgart C, Welling W, Hoppe MW, Freiwald J, Gokeler A. Angle-specific analysis of isokinetic quadriceps and hamstring torques and ratios in patients after ACL-reconstruction. BMC Sports Sci Med Rehabil. 2018;10:23. 10.1186/s13102-018-0112-6. 30534382 PMC6282246

[11] Fischer F, Fink C, Herbst E, et al. Higher hamstring-to-quadriceps isokinetic strength ratio during the first post-operative months in patients with quadriceps tendon compared to hamstring tendon graft following ACL reconstruction. Knee Surg Sports Traumatol Arthrosc. 2018;26:418–25. 10.1007/s00167-017-4522-x. 28324151

[12] Read PJ, Trama R, Racinais S, McAuliffe S, Klauznicer J, Alhammoud M. Angle specific analysis of hamstrings and quadriceps isokinetic torque identify residual deficits in soccer players following ACL reconstruction: a longitudinal investigation. J Sports Sci. 2022;40:871–7. 10.1080/02640414.2021.2022275. 34983321

[13] Maniar N, Cole MH, Bryant AL, Opar DA. Muscle force contributions to anterior cruciate ligament loading. Sports Med. 2022;52:1737–50. 10.1007/s40279-022-01674-3. 35437711 PMC9325827

[14] Kellis E. Quantification of quadriceps and hamstring antagonist activity. Sports Med. 1998;25:37–62. 10.2165/00007256-199825010-00004. 9458526

[15] Aagaard P, Simonsen EB, Magnusson SP, Larsson B, Dyhre-Poulsen P. A new concept for isokinetic hamstring:Quadriceps muscle strength ratio. Am J Sports Med. 1998;26:231–7. 10.1177/03635465980260021201. 9548116

[16] DeMorat G, Weinhold P, Blackburn T, Chudik S, Garrett W. Aggressive quadriceps loading can induce noncontact anterior cruciate ligament injury. Am J Sports Med. 2004;32:477–83. 10.1177/0363546503258928. 14977677

[17] Shelburne KB, Torry MR, Pandy MG. Muscle, ligament, and joint-contact forces at the knee during walking. Med Sci Sports Exerc. 2005;37:1948–56. 10.1249/01.mss.0000180404.86078.ff. 16286866

[18] Peel SA, Schroeder LE, Weinhandl JT. Lower extremity muscle contributions to ACL loading during a stop-jump task. J Biomech. 2021;121:110426. 10.1016/j.jbiomech.2021.110426. 33873112

[19] Toumi H, Poumarat G, Benjamin M, Best TM, F’Guyer S, Fairclough J. New insights into the function of the vastus medialis with clinical implications. Med Sci Sports Exerc. 2007;39:1153–9. 10.1249/01.mss.0b013e31804ec08d. 17596784

[20] Jeong J, Choi DH, Shin CS. Association between the medial-lateral quadriceps and hamstring muscle thickness and the knee kinematics and kinetics during single-leg landing. Sports Health. 2023;15:519–26. 10.1177/19417381231152476. 36856193 PMC10293562

[21] Achenbach L, Bloch H, Klein C, et al. Four distinct patterns of anterior cruciate ligament injury in women’s professional football (soccer): a systematic video analysis of 37 match injuries. Br J Sports Med. 2024;58:709–16. 10.1136/bjsports-2023-107113. 38684328 PMC11228206

[22] Ando R, Suzuki Y. Positive relationship between passive muscle stiffness and rapid force production. Hum Mov Sci. 2019;66. 10.1016/j.humov.2019.05.002. :285 – 91. 31082668

[23] Ema R. Association between elastography-assessed muscle mechanical properties and high-speed dynamic performance. Eur J Sport Sci. 2023;23:1233–9. 10.1080/17461391.2022.2097129. 35771491

[24] Hirata K, Ito M, Nomura Y, et al. Muscle quality indices separately associate with joint-level power-related measures of the knee extensors in older males. Eur J Appl Physiol. 2022;122:2271–81. 10.1007/s00421-022-05005-2. 35849183 PMC9463346

[25] Wilhelm EN, Rech A, Minozzo F, Radaelli R, Botton CE, Pinto RS. Relationship between quadriceps femoris echo intensity, muscle power, and functional capacity of older men. Age (Dordr). 2014;36:9625. 10.1007/s11357-014-9625-4. 24515898 PMC4082605

[26] Akagi R, Ichikawa S, Tanaka M, et al. Comparison of hamstring functional and stiffness characteristics between adolescent female soccer players and age-matched controls: a cross-sectional study. Res Q Exerc Sport. 2026;97:310–20. 10.1080/02701367.2025.2589333. 41397690

[27] Hirata K, Yamadera R, Akagi R. Can static stretching reduce stiffness of the triceps surae in older men? Med Sci Sports Exerc. 2020;52:673–9. 10.1249/MSS.0000000000002186. 31652247 PMC7034366

[28] Ward SR, Lieber RL. Density and hydration of fresh and fixed human skeletal muscle. J Biomech. 2005;38:2317–20. 10.1016/j.jbiomech.2004.10.001. 16154420

[29] Nordez A, Hug F. Muscle shear elastic modulus measured using supersonic shear imaging is highly related to muscle activity level. J Appl Physiol (1985). 2010;108:1389–94. 10.1152/japplphysiol.01323.2009. 20167669

[30] Cohen J. Statistical power analysis for the behavioral sciences. 2nd ed. New York: Lawrence Erlbaum Associates; 1988.

[31] Bakeman R. Recommended effect size statistics for repeated measures designs. Behav Res Methods. 2005;37:379–84. 10.3758/BF03192707. 16405133

[32] Olejnik S, Algina J. Generalized eta and omega squared statistics: measures of effect size for some common research designs. Psychol Methods. 2003;8:434–47. 10.1037/1082-989X.8.4.434. 14664681

[33] Lees A, Asai T, Andersen TB, Nunome H, Sterzing T. The biomechanics of kicking in soccer: a review. J Sports Sci. 2010;28:805–17. 10.1080/02640414.2010.481305. 20509089

[34] Kellis E, Katis A. The relationship between isokinetic knee extension and flexion strength with soccer kick kinematics: an electromyographic evaluation. J Sports Med Phys Fit. 2007;47:385–94. 18091676

[35] França C, Martins F, Przednowek K, et al. Knee and hip muscle strength of male soccer players from different competitive levels. J Hum Kinet. 2024;93:17–27. 10.5114/jhk/185217. 39132414 PMC11307174

[36] Cometti G, Maffiuletti NA, Pousson M, Chatard JC, Maffulli N. Isokinetic strength and anaerobic power of elite, subelite and amateur French soccer players. Int J Sports Med. 2001;22:45–51. 10.1055/s-2001-11331. 11258641

[37] Vargas VZ, Motta C, Peres BA, Vancini RL, De Andre Barbosa CAB, Andrade MS. Knee isokinetic muscle strength and balance ratio in female soccer players of different age groups: a cross-sectional study. Phys Sportsmed. 2020;48:105–9. 10.1080/00913847.2019.1642808. 31307251

[38] Stock MS, Thompson BJ. Echo intensity as an indicator of skeletal muscle quality: applications, methodology, and future directions. Eur J Appl Physiol. 2021;121:369–80. 10.1007/s00421-020-04556-6. 33221942

[39] Cruz-Montecinos C, Pinto MD, Pinto RS. Sex differences in quantitative ultrasonographic measurements of the rectus femoris in children. J Anat. 2024;246:98–107. 10.1111/joa.14136. 39325922 PMC11684384

[40] Terzis G, Vekaki E, Papadopoulos C, Papadimas G, Stasinaki A-N. Muscle ultrasound echo intensity and fiber type composition in young females. J Funct Morphol Kinesiol. 2024;9:64. 10.3390/jfmk9020064. 38651422 PMC11036197

[41] García-Alonso Y, Alonso-Martínez AM, García-Hermoso A, Legarra-Gorgoñon G, Izquierdo M, Ramírez-Vélez R. Centile reference curves of the ultrasound-based characteristics of the rectus femoris muscle composition in children at 4–11 years old. Front Pediatr. 2023;11:1168253. 10.3389/fped.2023.1168253. 37635791 PMC10449539

[42] Chino K, Ohya T, Kato E, Suzuki Y. Muscle thickness and passive muscle stiffness in elite athletes: implications of the effect of long-term daily training on skeletal muscle. Int J Sports Med. 2018;39:218–24. 10.1055/s-0043-122737. 29365338

[43] Mercan N, Elmali N, Çitil S, Bilsel K, Tuncay İ. High hamstring stiffness and flexibility with comparable spinopelvic morphometry in amateur footballers: a multimodal study. BMC Sports Sci Med Rehabil. 2025;17:219. 10.1186/s13102-025-01265-5. 40739273 PMC12308992

[44] Taş S, Özkan Ö, Karaçoban L, Dönmez G, Çetin A, Korkusuz F. Knee muscle and tendon stiffness in professional soccer players: a shear-wave elastography study. J Sports Med Phys Fit. 2020;60:276–81. 10.23736/S0022-4707.19.09938-9. 31663309

[45] Ramos AP, de Mesquita RS, Migliorini F, Maffulli N, Okubo R. FIFA 11 + KIDS in the prevention of soccer injuries in children: a systematic review. J Orthop Surg Res. 2024;19:413. 10.1186/s13018-024-04876-9. 39026353 PMC11264619

