# Supplementary material for: Muscular profiles of adolescent female soccer players: a cross-sectional comparison of hamstring-to-quadriceps and vastus medialis-to-vastus lateralis characteristics
Source: J Orthop Surg Res. 2026 Jun 23;21:538. doi: 10.1186/s13018-026-07063-0 (PMC13560221; doi:10.1186/s13018-026-07063-0)
Supplement: Supplementary file 1 [file 13018_2026_7063_MOESM1_ESM.docx]

**Table S1.** Statistical outcomes of the three-way mixed-design analysis of variance (ANOVA) for isometric knee flexor (KF) and extensor (KE) peak torque and isometric hamstrings-to-quadriceps torque ratio (H:Q ratio)

| parameter | ANOVA effects | *F* | *P* | *η*_G_^2^ |  |
| --- | --- | --- | --- | --- | --- |
| KF peak torque | interaction |  |  |  |  |
|  | leg × age × experience | *F*[1,93] = 4.169 | 0.044 | 0.002 | [trivial] |
|  | simple interaction |  |  |  |  |
|  | D: age × experience | *F*[1,93] = 0.966 | 0.328 |  |  |
|  | ND: age × experience | *F*[1,93] = 5.525 | 0.021 |  |  |
|  | simple main effect |  |  |  |  |
|  | D: age | *F*[1,93] = 27.410 | <0.001 |  |  |
|  | D: experience | *F*[1,93] = 24.168 | <0.001 |  |  |
|  | simple-simple main effect |  |  |  |  |
|  | ND: soccer: age | *F*[1,93] = 42.991 | <0.001 |  |  |
|  | ND: control: age | *F*[1,93] = 3.084 | 0.082 |  |  |
|  | ND: older: experience | *F*[1,93] = 44.851 | <0.001 |  |  |
|  | ND: younger: experience | *F*[1,93] = 3.972 | 0.049 |  |  |
|  |  |  |  |  |  |
| KE peak torque | interaction |  |  |  |  |
|  | leg × age × experience | *F*[1,93] = 0.227 | 0.635 | <0.001 | [trivial] |
|  | leg × age | *F*[1,93] = 0.627 | 0.431 | <0.001 | [trivial] |
|  | leg × experience | *F*[1,93] = 0.007 | 0.933 | <0.001 | [trivial] |
|  | age × experience | *F*[1,93] = 2.528 | 0.115 | 0.016 | [small] |
|  | main effect |  |  |  |  |
|  | leg | *F*[1,93] = 10.325 | 0.002 | 0.006 | [trivial] |
|  | age | *F*[1,93] = 39.649 | <0.001 | 0.247 | [large] |
|  | experience | *F*[1,93] = 16.846 | <0.001 | 0.105 | [medium] |
|  |  |  |  |  |  |
| isometric H:Q ratio | interaction |  |  |  |  |
|  | leg × age × experience | *F*[1,93] = 2.000 | 0.161 | 0.005 | [trivial] |
|  | leg × age | *F*[1,93] = 0.047 | 0.829 | <0.001 | [trivial] |
|  | leg × experience | *F*[1,93] = 0.037 | 0.848 | <0.001 | [trivial] |
|  | age × experience | *F*[1,93] = 0.035 | 0.852 | <0.001 | [small] |
|  | main effect |  |  |  |  |
|  | leg | *F*[1,93] = 2.117 | 0.149 | 0.005 | [trivial] |
|  | age | *F*[1,93] = 1.796 | 0.186 | 0.014 | [small] |
|  | experience | *F*[1,93] = 4.906 | 0.029 | 0.038 | [small] |

Values include *F*-statistics with degrees of freedom [1, 93], *P*-values, and generalized eta squared (*η*_G_^2^) for the KF and KE peak torque and the isometric H:Q ratio. The three-way ANOVA was performed with leg dominance (dominant vs. non-dominant) as a within-subject factor, and age (older vs. younger) and experience (soccer vs. control) as between-subject factors. Effect size magnitudes are interpreted based on Cohen’s benchmarks: trivial (< 0.010), small (0.010–0.059), medium (0.060–0.139), and large (≥ 0.140).
