# Supplementary material for: Muscular profiles of adolescent female soccer players: a cross-sectional comparison of hamstring-to-quadriceps and vastus medialis-to-vastus lateralis characteristics
Source: J Orthop Surg Res. 2026 Jun 23;21:538. doi: 10.1186/s13018-026-07063-0 (PMC13560221; doi:10.1186/s13018-026-07063-0)
Supplement: Supplementary file 2 [file 13018_2026_7063_MOESM2_ESM.docx]

**Table S2.** Statistical outcomes of the three-way mixed-design analysis of variance (ANOVA) for isotonic knee flexor (KF) and extensor (KE) peak power and isotonic hamstrings-to-quadriceps power ratio (H:Q ratio)

| parameter | ANOVA effects | *F* | *P* | *η*_G_^2^ |  |
| --- | --- | --- | --- | --- | --- |
| KF peak power | interaction |  |  |  |  |
|  | leg × age × experience | *F*[1,93] = 0.178 | 0.674 | <0.001 | [trivial] |
|  | leg × age | *F*[1,93] = 0.407 | 0.525 | <0.001 | [trivial] |
|  | leg × experience | *F*[1,93] = 0.629 | 0.430 | 0.001 | [trivial] |
|  | age × experience | *F*[1,93] = 1.628 | 0.205 | 0.009 | [trivial] |
|  | main effect |  |  |  |  |
|  | leg | *F*[1,93] = 1.526 | 0.220 | 0.002 | [trivial] |
|  | age | *F*[1,93] = 18.016 | <0.001 | 0.098 | [medium] |
|  | experience | *F*[1,93] = 50.831 | <0.001 | 0.276 | [large] |
|  |  |  |  |  |  |
| KE peak power | interaction |  |  |  |  |
|  | leg × age × experience | *F*[1,93] = 0.127 | 0.723 | <0.001 | [trivial] |
|  | leg × age | *F*[1,93] = 0.405 | 0.526 | <0.001 | [trivial] |
|  | leg × experience | *F*[1,93] = 0.876 | 0.352 | 0.001 | [trivial] |
|  | age × experience | *F*[1,93] = 0.913 | 0.342 | 0.006 | [trivial] |
|  | main effect |  |  |  |  |
|  | leg | *F*[1,93] = 51.600 | <0.001 | 0.034 | [small] |
|  | age | *F*[1,93] = 25.876 | <0.001 | 0.177 | [large] |
|  | experience | *F*[1,93] = 16.771 | <0.001 | 0.115 | [medium] |
|  |  |  |  |  |  |
| isotonic H:Q ratio | interaction |  |  |  |  |
|  | leg × age × experience | *F*[1,93] < 0.001 | 0.998 | <0.001 | [trivial] |
|  | leg × age | *F*[1,93] = 0.487 | 0.487 | 0.001 | [trivial] |
|  | leg × experience | *F*[1,93] = 3.710 | 0.057 | 0.011 | [small] |
|  | age × experience | *F*[1,93] = 0.070 | 0.792 | <0.001 | [trivial] |
|  | main effect |  |  |  |  |
|  | leg | *F*[1,93] = 35.353 | <0.001 | 0.095 | [medium] |
|  | age | *F*[1,93] = 4.358 | 0.040 | 0.031 | [small] |
|  | experience | *F*[1,93] = 3.385 | 0.069 | 0.024 | [small] |

Values include *F*-statistics with degrees of freedom [1, 93], *P*-values, and generalized eta squared (*η*_G_^2^) for the KF and KE peak power and the isotonic H:Q ratio. The three-way ANOVA was performed with leg dominance (dominant vs. non-dominant) as a within-subject factor, and age (older vs. younger) and experience (soccer vs. control) as between-subject factors. Effect size magnitudes are interpreted based on Cohen’s benchmarks: trivial (< 0.010), small (0.010–0.059), medium (0.060–0.139), and large (≥ 0.140).
