# Supplementary material for: Muscular profiles of adolescent female soccer players: a cross-sectional comparison of hamstring-to-quadriceps and vastus medialis-to-vastus lateralis characteristics
Source: J Orthop Surg Res. 2026 Jun 23;21:538. doi: 10.1186/s13018-026-07063-0 (PMC13560221; doi:10.1186/s13018-026-07063-0)
Supplement: Supplementary file 3 [file 13018_2026_7063_MOESM3_ESM.docx]

**Table S3.** Statistical outcomes of the three-way mixed-design analysis of variance (ANOVA) for vastus medialis (VM) and vastus lateralis (VL) echo intensity and VM:VL echo intensity ratio

| parameter | ANOVA effects | *F* | *P* | *η*_G_^2^ |  |
| --- | --- | --- | --- | --- | --- |
| VM echo intensity | interaction |  |  |  |  |
|  | leg × age × experience | *F*[1,93] = 1.658 | 0.201 | 0.003 | [trivial] |
|  | leg × age | *F*[1,93] = 0.398 | 0.529 | 0.001 | [trivial] |
|  | leg × experience | *F*[1,93] = 2.528 | 0.115 | 0.004 | [trivial] |
|  | age × experience | *F*[1,93] = 0.168 | 0.683 | 0.001 | [trivial] |
|  | main effect |  |  |  |  |
|  | leg | *F*[1,93] = 6.753 | 0.011 | 0.010 | [small] |
|  | age | *F*[1,93] = 14.581 | <0.001 | 0.101 | [medium] |
|  | experience | *F*[1,93] = 14.523 | <0.001 | 0.101 | [medium] |
|  |  |  |  |  |  |
| VL echo intensity | interaction |  |  |  |  |
|  | leg × age × experience | *F*[1,93] = 0.938 | 0.335 | 0.001 | [trivial] |
|  | leg × age | *F*[1,93] = 0.006 | 0.936 | <0.001 | [trivial] |
|  | leg × experience | *F*[1,93] = 0.682 | 0.411 | 0.001 | [trivial] |
|  | age × experience | *F*[1,93] = 0.030 | 0.864 | <0.001 | [trivial] |
|  | main effect |  |  |  |  |
|  | leg | *F*[1,93] = 0.792 | 0.376 | 0.001 | [trivial] |
|  | age | *F*[1,93] = 18.579 | <0.001 | 0.138 | [medium] |
|  | experience | *F*[1,93] = 10.150 | 0.002 | 0.075 | [medium] |
|  |  |  |  |  |  |
| VM:VL echo intensity ratio | interaction |  |  |  |  |
|  | leg × age × experience | *F*[1,93] = 2.337 | 0.130 | 0.009 | [trivial] |
|  | leg × age | *F*[1,93] = 0.181 | 0.672 | 0.001 | [trivial] |
|  | leg × experience | *F*[1,93] = 0.501 | 0.481 | 0.002 | [trivial] |
|  | age × experience | *F*[1,93] = 0.081 | 0.777 | 0.001 | [trivial] |
|  | main effect |  |  |  |  |
|  | leg | *F*[1,93] = 6.071 | 0.016 | 0.022 | [small] |
|  | age | *F*[1,93] = 0.291 | 0.591 | 0.002 | [trivial] |
|  | experience | *F*[1,93] = 0.598 | 0.441 | 0.004 | [trivial] |

Values include *F*-statistics with degrees of freedom [1, 93], *P*-values, and generalized eta squared (*η*_G_^2^) for the VM and VL echo intensity and the VM:VL ratio. The three-way ANOVA was performed with leg dominance (dominant vs. non-dominant) as a within-subject factor, and age (older vs. younger) and experience (soccer vs. control) as between-subject factors. Effect size magnitudes are interpreted based on Cohen’s benchmarks: trivial (< 0.010), small (0.010–0.059), medium (0.060–0.139), and large (≥ 0.140).
