# Supplementary material for: Muscular profiles of adolescent female soccer players: a cross-sectional comparison of hamstring-to-quadriceps and vastus medialis-to-vastus lateralis characteristics
Source: J Orthop Surg Res. 2026 Jun 23;21:538. doi: 10.1186/s13018-026-07063-0 (PMC13560221; doi:10.1186/s13018-026-07063-0)
Supplement: Supplementary file 4 [file 13018_2026_7063_MOESM4_ESM.docx]

**Table S4.** Statistical outcomes of the three-way mixed-design analysis of variance (ANOVA) for vastus medialis (VM) and vastus lateralis (VL) shear modulus and VM:VL shear modulus ratio

| parameter | ANOVA effects | *F* | *P* | *η*_G_^2^ |  |
| --- | --- | --- | --- | --- | --- |
| VM shear modulus | interaction |  |  |  |  |
|  | leg × age × experience | *F*[1,93] = 2.088 | 0.152 | 0.004 | [trivial] |
|  | leg × age | *F*[1,93] = 3.134 | 0.080 | 0.007 | [trivial] |
|  | leg × experience | *F*[1,93] = 0.148 | 0.701 | <0.001 | [trivial] |
|  | age × experience | *F*[1,93] = 1.643 | 0.203 | 0.011 | [small] |
|  | main effect |  |  |  |  |
|  | leg | *F*[1,93] = 3.418 | 0.068 | 0.007 | [trivial] |
|  | age | *F*[1,93] = 1.362 | 0.246 | 0.009 | [trivial] |
|  | experience | *F*[1,93] = 24.456 | <0.001 | 0.161 | [large] |
|  |  |  |  |  |  |
| VL shear modulus | interaction |  |  |  |  |
|  | leg × age × experience | *F*[1,93] = 0.006 | 0.936 | <0.001 | [trivial] |
|  | leg × age | *F*[1,93] = 0.058 | 0.809 | <0.001 | [trivial] |
|  | leg × experience | *F*[1,93] = 0.537 | 0.465 | 0.001 | [trivial] |
|  | age × experience | *F*[1,93] = 1.063 | 0.305 | 0.008 | [trivial] |
|  | main effect |  |  |  |  |
|  | leg | *F*[1,93] = 8.482 | 0.004 | 0.018 | [small] |
|  | age | *F*[1,93] = 0.057 | 0.812 | <0.001 | [trivial] |
|  | experience | *F*[1,93] = 15.238 | <0.001 | 0.110 | [medium] |
|  |  |  |  |  |  |
| VM:VL shear modulus ratio | interaction |  |  |  |  |
|  | leg × age × experience | *F*[1,93] = 0.881 | 0.350 | 0.002 | [trivial] |
|  | leg × age | *F*[1,93] = 1.580 | 0.212 | 0.004 | [trivial] |
|  | leg × experience | *F*[1,93] = 0.612 | 0.436 | 0.002 | [trivial] |
|  | age × experience | *F*[1,93] < 0.001 | 0.994 | <0.001 | [trivial] |
|  | main effect |  |  |  |  |
|  | leg | *F*[1,93] = 10.910 | 0.001 | 0.029 | [small] |
|  | age | *F*[1,93] = 0.304 | 0.583 | 0.002 | [trivial] |
|  | experience | *F*[1,93] = 0.001 | 0.975 | <0.001 | [trivial] |

Values include *F*-statistics with degrees of freedom [1, 93], *P*-values, and generalized eta squared (*η*_G_^2^) for the VM and VL shear modulus and the VM:VL ratio. The three-way ANOVA was performed with leg dominance (dominant vs. non-dominant) as a within-subject factor, and age (older vs. younger) and experience (soccer vs. control) as between-subject factors. Effect size magnitudes are interpreted based on Cohen’s benchmarks: trivial (< 0.010), small (0.010–0.059), medium (0.060–0.139), and large (≥ 0.140).
